# Outage Analysis of the Power Splitting Based Underlay Cooperative Cognitive Radio Networks

**DOI:** 10.3390/s21227653

**Published:** 2021-11-18

**Authors:** Phu Tran Tin, Van-Duc Phan, Tan N. Nguyen, Lam-Thanh Tu, Bui Vu Minh, Miroslav Voznak, Peppino Fazio

**Affiliations:** 1 Faculty of Electronics Technology, Industrial University of Ho Chi Minh City, Ho Chi Minh City 700000, Vietnam; phutrantin@iuh.edu.vn; 2 Faculty of Automobile Technology, Van Lang University, Ho Chi Minh City 700000, Vietnam; duc.pv@vlu.edu.vn; 3 Communication and Signal Processing Research Group, Faculty of Electrical and Electronics Engineering, Ton Duc Thang University, Ho Chi Minh City 700000, Vietnam; 4 Institute XLIM, University of Poitiers, 86000 Poitiers, France; lamthanh0@gmail.com; 5 Faculty of Automotive, Mechanical, Electrical and Electronic Engineering, Nguyen Tat Thanh University, Ho Chi Minh City 700000, Vietnam; bvminh@ntt.edu.vn; 6 Faculty of Electrical Engineering and Computer Science, VSB-Technical University of Ostrava, 708 00 Ostrava, Czech Republic; miroslav.voznak@vsb.cz (M.V.); pepfaz77@gmail.com (P.F.); 7 Department of Molecular Sciences and Nanosystems, Ca’ Foscari University of Venice, Via Torino 155, 30123 Venezia VE, Italy

**Keywords:** decode–and–forward, outage probability, relay selection, cognitive radio network, SWIPT

## Abstract

In the present paper, we investigate the performance of the simultaneous wireless information and power transfer (SWIPT) based cooperative cognitive radio networks (CCRNs). In particular, the outage probability is derived in the closed-form expressions under the opportunistic partial relay selection. Different from the conventional CRNs in which the transmit power of the secondary transmitters count merely on the aggregate interference measured on the primary networks, the transmit power of the SWIPT-enabled transmitters is also constrained by the harvested energy. As a result, the mathematical framework involves more correlated random variables and, thus, is of higher complexity. Monte Carlo simulations are given to corroborate the accuracy of the mathematical analysis and to shed light on the behavior of the OP with respect to several important parameters, e.g., the transmit power and the number of relays. Our findings illustrate that increasing the transmit power and/or the number of relays is beneficial for the outage probability.

## 1. Introduction

Cognitive radio networks (CRNs) are considered one of the most effective solutions to overcome the scarcity of the frequency spectrum [1,2]. The principal idea of CRNs is to permit unlicensed users to concurrently operate with licensed users while strictly guaranteeing the quality-of-service (QoS) of primary users. To realize such networks two popular protocols are proposed in the literature, namely, the overlay and underlay protocols [3,4]. The former allows secondary devices to opportunistically occupy the temporarily unused spectrum in terms of space, time, and frequency. Regarding the underlay protocol, on the other hand, secondary users are always granted permission to access the licensed spectrum provided that the aggregate interference created by secondary networks measured on the primary devices is below the predefined threshold. Compared to the overlay protocol, the underlay protocol is preferable due to its high availability and, thus, is suitable for urgent services, video conferences, video gaming, and so forth. Nevertheless, the cons of this protocol are that it does not support a long transmission and/or high-quality services owing to the low transmit power to avoid exceeding the interference threshold of the primary networks. As a consequence, in order to employ the underlay CRNs in practice, the combination with other techniques is necessary. Fortunately, relaying or cooperative communications is a good complement to the underlay CRNs [5,6,7]. More precisely, cooperative communications networks are wireless networks where one or several relays are deployed among end-users to help them exchange information. With the help of the relay, the transmission distance is dramatically decreased, improving the system performance. Additionally, relaying technology has also been proven to be an effective way to extend the coverage area. The spectral efficiency and QoS issues can be addressed in a straightforward manner by employing the cooperative cognitive radio networks (CCRNs) [8,9]. Nonetheless, there still exists an urgent issue, which is to improve the energy efficiency of wireless networks. The problem escalates seriously in either the Internet of Things (IoTs), low power wide area networks (LPWAN) [10], or 5G and beyond networks owing to the exponential growth of wireless-connected devices accompanied by their power-hungry applications. In fact, improving energy efficiency is one of the highest priorities in wireless communications. Fortunately, simultaneous wireless information and power transfer (SWIPT) has recently been proposed to deal with this issue [11,12,13]. In particular, SWIPT transmits both information and energy on the same carrier frequency, thus boosting both the spectral and energy efficiency. Consequently, in the present paper, we investigate the performance of the SWIPT-based cooperative cognitive radio networks (CCRNs) with the help of multiple relays. Before highlighting our novel contributions, the state-of-the-art is given as follows.

The performance of CCRNs networks was studied widely in [14,15,16,17,18,19,20]. Specifically, the outage probability (OP) of the cognitive radio non-orthogonal multiple access (NOMA) networks was derived in [14]. The authors in [15], on the other hand, investigated the secrecy performance of the underlay cooperative multihop CRNs. In particular, the secrecy outage probability (SOP) was derived in the approximated closed-form expression. Lee et al. in [16] addressed the minimization of the number of feedback bits required in order to satisfy the QoS in both primary and secondary networks. The optimal power allocation of non-orthogonal amplify–and–forward (AF) relaying of underlay CRNs was provided in [17] to maximize the system throughput. In [18], the capacity of voice over IP (VoIP) in CRNs was analyzed and maximized by modelling the VoIP traffic and channel coefficients as a Markov-modulated Poisson process. Liu et al. in [19] investigated the spectrum sensing problem in the full-duplex cooperative spectrum sensing CRNs. It was noted that spectrum sensing is important in overlay CRNs to guarantee the opportunistic access of secondary devices while not interrupting the primary device’s transmission.

Meanwhile, the performance of SWIPT-enabled networks was investigated in [21,22,23,24,25,26,27,28,29]. The energy-efficient optimization of SWIPT-assisted relaying networks was addressed in [21]. Tan et al. in [22] derived the OP and ergodic capacity of the power splitting (PS) based relaying networks under the asymmetric channel, i.e., the Nakagami-*m* and Rayleigh channels. The error probability and outage probability of SWIPT-based NOMA networks were derived in [23]. In particular, the pairwise error probability was computed in closed-form expression in [23]. The asymptotic framework under a high SNRs regime was provided as well. The work in [24], differently, derived the symbol error rate (SER) of SWIPT-enabled relaying networks, where the noncoherent modulation was employed in place of the conventional phase-shift keying (PSK) and/or quadrature amplitude modulation (QAM). The authors in [25] also addressed the noncoherent modulation. Specifically, this work first derived the moments and moment generating function (MGF) of the end-to-end (e2e) signal-to-noise ratios (SNRs). Based on the MGF, they then computed the outage probability, the amount of fading, and the system throughput. The authors in [26,27] dealt with the physical layer security (PLS) issue of the simultaneous wireless information and power transfer assisted relaying networks. Furthermore, the performance of SWIPT-based cellular networks with and without utilizing millimeter wave (mmWave) was provided in [28,29].

Despite the extensive study of either the CCRNs or the SWIPT-aided networks, the performance of SWIPT-enabled underlay CCRNs is still in the infancy stage. In particular, there only a few works addressing this combination [30,31,32,33]. Specifically, Prathima et al. in [30] studied the performance of the primary networks with the help of the secondary users that also act as the relay of primary users. This work, however, concentrated on the performance of primary networks. Moreover, it solely considered two relay nodes instead of the general scenario. The work in [31], diversely, addressed the secrecy performance of the SWIPT-assisted cognitive relaying networks. The power allocation and transceiver design were investigated in [32].

In this paper, different from the abovementioned works, we focus on the performance of the secondary networks as well as the reliability of SWIPT-based underlay cooperative cognitive radio networks with regard to transceiver design, power allocation, and physical layer security. In particular, the principal novel contributions are summarized as follows:

- We consider a single-input single-output (SISO) underlay cooperative cognitive radio network with the assistance of multiple relays. Additionally, the transmit power of the relay nodes relies only on the harvested energy from the transmitter S. The partial relay selection is adopted to both enhance the system performance and reduce the complexity compared to the fully relay selection.
- Different from the conventional underlay CRNs where the transmit power of the secondary transmitter considers merely the interference power, the transmit power of the considered networks is constrained by both the interference power and the harvested energy. The mathematical framework, thus, is of higher complexity owing to dealing with more correlated random variables. Nonetheless, we are still able to derive the outage probability in the closed-form expressions.
- Simulation results are presented to corroborate the exactness of our analysis and to identify the behavior of OP with respect to several important parameters, namely, the transmit power, the number of relays, the power splitting ratio, and so on. Our findings show that both increasing the transmit power and number of relays are beneficial to the OP. Additionally, an optimal value of the power splitting ratio exists that minimizes the OP.

The remainder of this paper is organized as follows. The system model is given in Section 2. The derivation of the OP is provided in Section 3. Numerical results are shown in Section 4. Section 5 concludes the paper.

## 2. System Model

**Figure 1:**
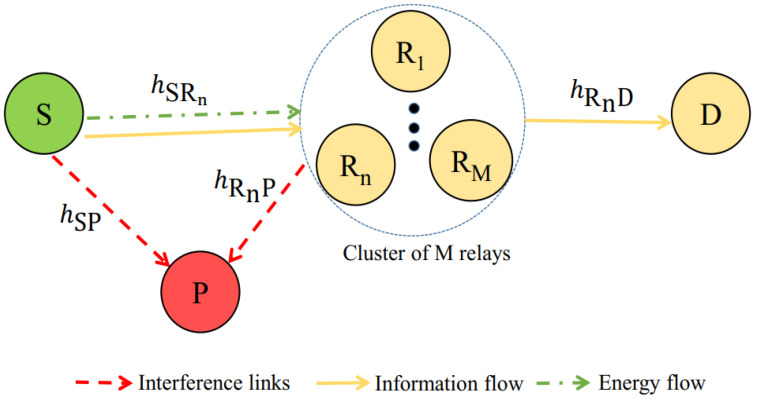
SWIPT-based cognitive radio relaying networks.

Let us consider SWIPT-based underlay cognitive radio networks as shown in Figure 1. In particular, the secondary networks comprise one source node denoted by S, one destination denoted by D, and *M* relay nodes denoted by $R_{i},i\in \left\{1,\ldots ,M\right\}$, while the primary networks are represented by a primary receiver denoted by P. Here, P measures the aggregate interference created by the secondary networks on the primary networks.

### 2.1. Channel Modeling

Considering a generic transmission from node X to node Y, the channel coefficients denoted by $h_{XY}$, $X\in \left\{S,R_{i}\right\}$, $Y\in \left\{R_{i},D\right\}$ are followed by a Rayleigh distribution. As a result, the channel gain denoted by $\gamma_{XY}={\left|h_{XY}\right|}^{2}$ is followed by an exponential distribution with parameter $\lambda_{XY}$ whose cumulative distribution function (CDF) and probability density function (PDF) are given as follows [6]:

(1) $$\begin{array}{c} F_{X}\left(x\right)=1-\exp\left(-\lambda_{XY}x\right), \end{array}$$

(2) $$\begin{array}{c} f_{X}\left(x\right)=\frac{\partial F_{X}\left(x\right)}{\partial x}=\lambda_{XY}\exp\left(-\lambda_{XY}x\right). \end{array}$$

Here $\lambda_{XY}$ is also the large-scale path loss from *X* to *Y* and is formulated as follows:

(3) $$\begin{array}{c} \lambda_{XY}={\left(d_{XY}\right)}^{\beta }, \end{array}$$

where $d_{XY}$ is the Euclidean distance between node *X* and *Y* and $\beta \in \;\left(2,\ldots ,6\right)$ is the path loss exponent. Additionally, the block fading is taken into consideration in this work, hence the channel coefficients remain constants for the whole transmission *T* and change independently between each transmission.

### 2.2. PS-Based Relaying Networks

In this work, we adopt the power-splitting (PS) protocol at the relay node. To be more precise, the received power at R is divided into two separate parts according to the power-splitting ratio ρ, 0<ρ<1, i.e., one is put into the energy harvester and another goes to the information decoder. ρ takes into account all loss introduced by the energy harvesting receiver, e.g., noise introduced by the received antenna, loss due to the converting RF-to-DC circuit, and so on [34,35]. Additionally, to realize the power-splitting protocol, each SWIPT-enabled receiver needs to be equipped with a power splitter to split the received power into two parts. The first part is sent to the conventional information decoding circuit, and the remaining part is sent to the energy harvesting circuit [35,36].

### 2.3. Opportunistic Partial Relaying (OPR) Protocol

In this paper, the opportunistic partial relaying (OPR) protocol is adopted. In particular, only the relay *n* denoted by R${}_{n}$, which has the highest channel gain from S to all relay nodes, is selected to help exchange information between S and D. Other relay nodes, as a result, keep silent in order to save energy consumption and avoid creating co-channel interference.

(4) $$\begin{array}{c} R_{n}\;\;:\;\;\gamma_{SR_{n}}=\underset{m=1,2,\ldots ,M}{\underbrace{\max}}\left\{\gamma_{SR_{m}}\right\} \end{array}$$

Compared with the scenario where all relays participate in the transmission, our adopted protocol is simpler since it does not require perfect channel state information (CSI) of all nodes of the networks at the destination and perfect synchronization among relays [37,38,39]. To be more precise, the adopted OPR protocol can be employed as follows. Each relay is equipped with a timer, and the value of the timer is set inversely with the channel gain from S to relay. Thus, the best relay is the one having the smallest timer. When the timer ends, the best relay forwards the source’s signal to the destination. Other relays sense the availability of the medium and keep silent once the medium is occupied.

### 2.4. Information Transmission

**Figure 2:**
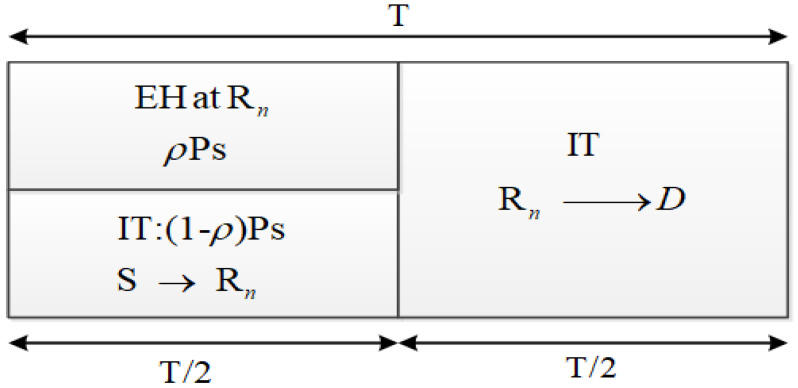
Energy harvesting (EH) and information transmission (IT) processes. EH takes place only in the first half of the transmission duration, while IT takes place during the whole transmission duration.

The whole transmission takes place in two phases. In the first phase, source S broadcasts its signals to all relay nodes. Here, we assume that the direct channel between S and D does not exist due to the long transmission distance and deep fades; thus, destination D does not receive the broadcast signal from S. Although all relays are received signals sent by S, only relay R${}_{n}$ is selected to assist the transmission from S to D. The criteria for selecting R${}_{n}$ is given in Section 2.3. At relay R${}_{n}$, parts of the incoming signals are sent to the information decoder to decode the information sent by S and are given as

(5) $$\begin{array}{c} y_{R_{n}}=\sqrt{1-\rho }\sqrt{P_{S}}h_{SR_{n}}x_{S}+n_{R_{n}}, \end{array}$$

where $n_{R_{n}}$ is the additive white Gaussian noise (AWGN) at relay R${}_{n}$, which follows a complex Gaussian distribution with zero mean and $N_{0}$ variance, $n_{R_{n}}∼\mathrm{CN}\left(0,N_{0}\right)$; $x_{S}$ is the transmitted signal of S and $E\left\{{\left|x_{S}\right|}^{2}\right\}=1$; $E\left\{•\right\}$ is the expectation operator; and $P_{S}$ is the transmit power of S and is defined in the sequel. The remaining part of the incoming signals from S is put into the energy harvested receiver. The amount of harvested energy denoted by $E_{R_{n}}$ are then formulated as

(6) $$\begin{array}{c} E_{R_{n}}=\eta \rho \left(T/2\right)P_{S}{\left|h_{SR_{n}}\right|}^{2}, \end{array}$$

where η is the energy conversion coefficient [34,40]; the factor T/2 implies that the energy harvesting only takes place in half of the whole transmission procedure. At the end of the first phase, relay R${}_{n}$ decodes the information sent by S and forwards the re-encoded version to the destination D in the second phase. The whole transmission procedure is shown in Figure 2. The received signals at D is then formulated as

(7) $$\begin{array}{c} y_{D}=\sqrt{P_{R_{n}}}h_{R_{n}D}x_{R_{n}}+n_{D}, \end{array}$$

where $n_{D}$ is the AWGN noise at D with zero mean and $N_{0}$ variance; $x_{R_{n}}$ is the transmitted signals of the relay R${}_{n}$ with $E\left\{{\left|x_{R_{n}}\right|}^{2}\right\}=1$, and $P_{R_{n}}$ is the transmit power of R${}_{n}$ and is defined in Section 2.5. It is noted that the received signal at $R_{n}$ and D in Equations (5) and (7) is a function of the large-scale path loss via the channel coefficient $h_{SR_{n}}$ and $h_{R_{n}D}$, respectively. The signal-to-noise ratios at R${}_{n}$ and D are then formulated as follows:

(8) $$\begin{array}{cc} \gamma_{R_{n}}= & \frac{\left(1-\rho \right)\gamma_{SR_{n}}P_{S}}{N_{0}},\\ \gamma_{D}= & \frac{P_{R_{n}}{\left|h_{R_{n}D}\right|}^{2}}{N_{0}}. \end{array}$$

### 2.5. Transmit Power at Source and Relay Nodes

In the underlay cognitive radio networks, all secondary transmitters, i.e., the source node S and all relay R, have to adjust their transmit power to strictly satisfy the interference power threshold denoted by $I_{P}$ (in Watt) on the primary networks, i.e., the primary node P. As a result, the transmit power of S is then given as ([15], Equation (5))

(9) $$\begin{array}{c} P_{S}=\frac{I_{P}}{{\left|h_{\mathrm{SP}}\right|}^{2}}, \end{array}$$

Regarding the transmit power of R${}_{n}$, we have

(10) $$\begin{array}{c} P_{R_{n}}=\frac{I_{P}}{{\left|h_{R_{n}P}\right|}^{2}}, \end{array}$$

Additionally, the transmit power of the relay is also constrained by the amount of the harvested energy in the first phase and is formulated as ([22], Equation (2))

(11) $$\begin{array}{c} P_{R_{n}}=\frac{E_{R_{n}}}{T/2}=\eta \rho P_{S}{\left|h_{SR_{n}}\right|}^{2}. \end{array}$$

As a consequence, $P_{R_{n}}$ can be rewritten as follows

(12) $$\begin{array}{c} P_{R_{n}}=\min\left(\frac{I_{P}}{{\left|h_{R_{n}P}\right|}^{2}},\eta \rho P_{S}{\left|h_{SR_{n}}\right|}^{2}\right)=\min\left(\frac{I_{P}}{\gamma_{R_{n}P}},\eta \rho P_{S}\gamma_{SR_{n}}\right)\overset{\left(a\right)}{=}I_{P}\min\left(\frac{1}{\gamma_{R_{n}P}},\frac{\eta \rho \gamma_{SR_{n}}}{\gamma_{\mathrm{SP}}}\right), \end{array}$$

where $\left(a\right)$ is obtained by substituting $P_{S}$ in (9).

### 2.6. End-to-End Signal-to-Noise Ratios at D

Since the decode and forward (DF) protocol is employed, the e2e SNRs is then computed as

(13) $$\begin{array}{cc} \gamma_{e2e}= & \min\left\{\gamma_{R_{n}},\gamma_{D}\right\}\\ \overset{\left(a\right)}{=} & \Psi \min\left\{\frac{\left(1-\rho \right)\gamma_{SR_{n}}}{\gamma_{\mathrm{SP}}},\min\left(\frac{\gamma_{R_{n}D}}{\gamma_{R_{n}P}},\frac{\eta \rho \gamma_{SR_{n}}\gamma_{R_{n}D}}{\gamma_{\mathrm{SP}}}\right)\right\}, \end{array}$$

where $\Psi =\frac{I_{p}}{N_{0}}$; $\left(a\right)$ is held by substituting $P_{S}$ and $P_{R_{n}}$ in (9) and (12) into (8).

Through direct inspection (13), we observe that the e2e SNR of the considered system is more challenging than other work described in the literature. More precisely, the SNR is the composite of two minimum functions instead of only one. Additionally, the random variables inside these minimum functions are fully correlated as well. As a result, the proposed mathematical framework is novel and more complicated than others.

## 3. Outage Probability (OP) Analysis

In this section, we investigate one of the most important metrics of a wireless communications system, namely, the outage probability which measures the quality-of-service of the whole network. The OP referrs to the probability that the e2e SNRs at D is below a predefined threshold. Mathematically speaking, it is formulated as follows ([12], Equation (23)):

(14) $$\begin{array}{c} \mathrm{OP}=\mathrm{Pr}\left\{\gamma_{e2e}<\gamma_{th}\right\}=\mathrm{Pr}\left\{\min\left(\gamma_{R_{n}},\gamma_{D}\right)<\gamma_{th}\right\},=1-\mathrm{Pr}\left\{\gamma_{R_{n}}\geq \gamma_{th},\gamma_{D}\geq \gamma_{th}\right\}, \end{array}$$

where $\gamma_{th}=2^{2R}-1$, and R is the targeted rate [in bps/Hz]. In order to compute OP in (14), we first derive Lemma 1 as follows:

**Lemma** **1.**  *Given N independent and identically distributed (i.i.d.) exponential random variables (RVs) with parameters *Ω* denoted by $Y_{m},m\in \left\{1,\ldots ,N\right\}$. The CDF and PDF of the maximal RV denoted by $Y_{\max}=\max_{m\in \left\{1,\ldots ,N\right\}}\left\{Y_{m}\right\}$ are given as follows:*

(15) $$\begin{array}{cc} F_{Y_{\max}}\left(x\right)= & 1+\sum_{m=1}^{N}{\left(-1\right)}^{m}C_{N}^{m}\exp\left(-m\Omega x\right)\\ f_{Y_{\max}}\left(x\right)= & \Omega \sum_{m=1}^{N-1}{\left(-1\right)}^{m}C_{N-1}^{m}\exp\left(-\left(m+1\right)\Omega x\right) \end{array}$$

*where $C_{N}^{k}=\frac{N!}{k!(N-k)!}$ is the binomial coefficient.*

**Proof.**  Let us begin with the definition of the CDF as follows:

(16) $$\begin{array}{cc} F_{Y_{\max}}\left(x\right)= & \mathrm{Pr}\left\{Y_{\max}=\max_{m\in \left\{1,\ldots ,N\right\}}\left\{Y_{m}\right\}<x\right\}\\ \overset{\left(a\right)}{=} & \prod_{m=1}^{N}F_{Y_{m}}\left(x\right)\overset{\left(b\right)}{=}{\left(1-\exp\left(-\Omega x\right)\right)}^{N}\\ \overset{\left(c\right)}{=} & 1+\sum_{m=1}^{N}{\left(-1\right)}^{m}C_{N}^{m}\exp\left(-m\Omega x\right), \end{array}$$

where $\left(a\right)$ is held owing to the independence property between RVs; $\left(b\right)$ is attained by yielding the CDF of $Y_{m}$; and $\left(c\right)$ is achieved with the help of the binomial theorem. Taking the first-order derivative of the CDF with respect to *x*, we attain the PDF as follows:

(17) $$\begin{array}{cc} f_{Y_{\max}}\left(x\right)= & \frac{\partial F_{Y_{\max}}\left(x\right)}{\partial x}=\Omega \sum_{m=1}^{N-1}{\left(-1\right)}^{m}C_{N-1}^{m}\exp\left(-\left(m+1\right)\Omega x\right). \end{array}$$

We close the proof here. □

Next, the OP in (14) is rewritten as follows:

(18) $$\begin{array}{cc} \mathrm{OP}\overset{\left(a\right)}{=} & 1-\mathrm{Pr}\left\{\frac{\left(1-\rho \right)\gamma_{SR_{n}}\Psi }{\gamma_{\mathrm{SP}}}\geq \gamma_{th},\frac{\Psi \gamma_{R_{n}D}}{\gamma_{R_{n}P}}\geq \gamma_{th},\frac{\Psi \eta \rho \gamma_{SR_{n}}\gamma_{R_{n}D}}{\gamma_{\mathrm{SP}}}\geq \gamma_{th}\right\}\\ = & 1-\underset{\Xi }{\underbrace{\mathrm{Pr}\left\{X\geq \frac{\gamma_{th}}{(1-\rho )\Psi },\gamma_{R_{n}D}\geq \frac{\gamma_{th}\gamma_{R_{n}P}}{\Psi },X\geq \frac{\gamma_{th}}{\Psi \eta \rho \gamma_{R_{n}D}}\right\}}}, \end{array}$$

where $\left(a\right)$ is attained by substituting (13) into (14), $X=\frac{\gamma_{SR_{n}}}{\gamma_{\mathrm{SP}}}.$ Ξ in (18) can be calculated as follows:

(19) $$\begin{array}{cc} \Xi = & \underset{\Xi_{1}}{\underbrace{\mathrm{Pr}\left\{X\geq \frac{\gamma_{th}}{(1-\rho )\Psi },\gamma_{R_{n}D}\geq \frac{\gamma_{th}\gamma_{R_{n}P}}{\Psi },\frac{\gamma_{th}}{(1-\rho )\Psi }\geq \frac{\gamma_{th}}{\Psi \eta \rho \gamma_{R_{n}D}}\right\}}}\\ \; & +\underset{\Xi_{2}}{\underbrace{\mathrm{Pr}\left\{X\geq \frac{\gamma_{th}}{\Psi \eta \rho \gamma_{R_{n}D}},\gamma_{R_{n}D}\geq \frac{\gamma_{th}\gamma_{R_{n}P}}{\Psi },\frac{\gamma_{th}}{(1-\rho )\Psi }<\frac{\gamma_{th}}{\Psi \eta \rho \gamma_{R_{n}D}}\right\}}}. \end{array}$$

$\Xi_{1}$ in (19) can be split into two probabilities, i.e., $\Xi_{11}$ and $\Xi_{12}$, as follows:

(20) $$\begin{array}{cc} \Xi_{1}= & \mathrm{Pr}\left\{X\geq \frac{\gamma_{th}}{(1-\rho )\Psi },\gamma_{R_{n}D}\geq \frac{\gamma_{th}\gamma_{R_{n}P}}{\Psi },\gamma_{R_{n}D}\geq \frac{\left(1-\rho \right)}{\eta \rho }\right\}\\ = & \underset{\Xi_{11}}{\underbrace{\mathrm{Pr}\left\{X\geq \frac{\gamma_{th}}{(1-\rho )\Psi },\gamma_{R_{n}D}\geq \frac{\gamma_{th}\gamma_{R_{n}P}}{\Psi },\frac{\gamma_{th}\gamma_{R_{n}P}}{\Psi }\geq \frac{\left(1-\rho \right)}{\eta \rho }\right\}}}\\ \; & +\underset{\Xi_{12}}{\underbrace{\mathrm{Pr}\left\{X\geq \frac{\gamma_{th}}{(1-\rho )\Psi },\gamma_{R_{n}D}\geq \frac{\left(1-\rho \right)}{\eta \rho },\frac{\gamma_{th}\gamma_{R_{n}P}}{\Psi }<\frac{\left(1-\rho \right)}{\eta \rho }\right\}}}, \end{array}$$

where $\Xi_{11}$ is evaluated as

(21) $$\begin{array}{c} \Xi_{11}=\underset{P_{1}}{\underbrace{\mathrm{Pr}\left\{X\geq \frac{\gamma_{th}}{(1-\rho )\Psi }\right\}}}\times \underset{P_{2}}{\underbrace{\mathrm{Pr}\left\{\gamma_{R_{n}D}\geq \frac{\gamma_{th}\gamma_{R_{n}P}}{\Psi },\frac{\gamma_{th}\gamma_{R_{n}P}}{\Psi }\geq \frac{\left(1-\rho \right)}{\eta \rho }\right\}}}, \end{array}$$

Looking at (21), to compute $\Xi_{11}$, we first compute $P_{1}$ as follows:

(22) $$\begin{array}{cc} P_{1}= & 1-\mathrm{Pr}\left(\frac{\gamma_{SR_{n}}}{\gamma_{\mathrm{SP}}}<\frac{\gamma_{th}}{(1-\rho )\Psi }\right)=1-\int_{0}^{\infty }F_{\gamma_{SR_{n}}}\left(\frac{\gamma_{th}x}{(1-\rho )\Psi }\right)\times f_{\gamma_{\mathrm{SP}}}\left(x\right)dx,\\ \overset{\left(a\right)}{=} & \sum_{k=1}^{M}{\left(-1\right)}^{k+1}C_{M}^{k}\int_{0}^{\infty }\lambda_{\mathrm{SP}}\exp\left(-x\left[\frac{k\lambda_{\mathrm{SR}}\gamma_{th}}{(1-\rho )\Psi }+\lambda_{\mathrm{SP}}\right]\right)dx\\ = & \sum_{k=1}^{M}\frac{{\left(-1\right)}^{k+1}C_{M}^{k}\lambda_{\mathrm{SP}}\left(1-\rho \right)\Psi }{k\lambda_{\mathrm{SR}}\gamma_{th}+\lambda_{\mathrm{SP}}\left(1-\rho \right)\Psi }, \end{array}$$

where $\left(a\right)$ is achieved by utilizing Lemma 1. Next, $P_{2}$ is calculated as

(23) $$\begin{array}{cc} P_{2}= & \mathrm{Pr}\left\{\frac{(1-\rho )\Psi }{\eta \rho \gamma_{th}}\leq \gamma_{R_{n}P}\leq \frac{\gamma_{R_{n}D}\Psi }{\gamma_{th}}\right\}=\int_{\frac{(1-\rho )\Psi }{\eta \rho \gamma_{th}}}^{\infty }f_{\gamma_{R_{n}D}}\left(x\right)dx\int_{\frac{(1-\rho )\Psi }{\eta \rho \gamma_{th}}}^{\frac{x\Psi }{\gamma_{th}}}f_{\gamma_{R_{n}P}}\left(y\right)dy\\ = & \lambda_{\mathrm{RD}}\int_{\frac{(1-\rho )\Psi }{\eta \rho \gamma_{th}}}^{\infty }\exp\left(-\lambda_{\mathrm{RD}}x\right)\left\{\exp\left(-\frac{\left(1-\rho \right)\Psi \lambda_{\mathrm{RP}}}{\eta \rho \gamma_{th}}\right)-\exp\left(-\frac{x\Psi \lambda_{\mathrm{RP}}}{\gamma_{th}}\right)\right\}dx\\ = & \exp\left(-\frac{\left(1-\rho \right)\Psi \lambda_{\mathrm{RP}}}{\eta \rho \gamma_{th}}\right)\int_{\frac{(1-\rho )\Psi }{\eta \rho \gamma_{th}}}^{\infty }\lambda_{\mathrm{RD}}\exp\left(-\lambda_{\mathrm{RD}}x\right)dx\\ \; & -\lambda_{\mathrm{RD}}\int_{\frac{(1-\rho )\Psi }{\eta \rho \gamma_{th}}}^{\infty }\exp\left(-x\left[\lambda_{\mathrm{RD}}+\frac{\Psi \lambda_{\mathrm{RP}}}{\gamma_{th}}\right]\right)dx\\ = & \exp\left(-\frac{\left(1-\rho \right)\Psi \lambda_{\mathrm{RP}}}{\eta \rho \gamma_{th}}-\frac{\left(1-\rho \right)\Psi \lambda_{\mathrm{RD}}}{\eta \rho \gamma_{th}}\right)\\ \; & -\frac{\lambda_{\mathrm{RD}}}{\lambda_{\mathrm{RD}}+\frac{\Psi \lambda_{\mathrm{RP}}}{\gamma_{th}}}\exp\left(-\frac{(1-\rho )\Psi }{\eta \rho \gamma_{th}}\left[\lambda_{\mathrm{RD}}+\frac{\Psi \lambda_{\mathrm{RP}}}{\gamma_{th}}\right]\right), \end{array}$$

From (22) and (23), $\Xi_{11}$ in (21) is then computed as

(24) $$\begin{array}{cc} \Xi_{11}= & \sum_{k=1}^{M}\frac{{\left(-1\right)}^{k+1}C_{M}^{k}\lambda_{\mathrm{SP}}\left(1-\rho \right)\Psi }{k\lambda_{\mathrm{SR}}\gamma_{th}+\lambda_{\mathrm{SP}}\left(1-\rho \right)\Psi }\\ \; & \times \left[\exp\left(-\frac{\zeta \Psi \left[\lambda_{\mathrm{RP}}+\lambda_{\mathrm{RD}}\right]}{\gamma_{th}}\right)-\frac{\exp\left(-\frac{\zeta \Psi }{\gamma_{th}}\left[\lambda_{\mathrm{RD}}+\frac{\Psi \lambda_{\mathrm{RP}}}{\gamma_{th}}\right]\right)}{\left(1+\frac{\Psi \lambda_{\mathrm{RP}}}{\gamma_{th}\lambda_{\mathrm{RD}}}\right)}\right], \end{array}$$

where $\zeta =\frac{1-\rho }{\eta \rho }$. Having obtained the $\Xi_{11}$, we now move to $\Xi_{12}$. Let us first rewrite $\Xi_{12}$ as

(25) $$\begin{array}{cc} \Xi_{12}= & \mathrm{Pr}\left\{X\geq \frac{\gamma_{th}}{(1-\rho )\Psi },\gamma_{R_{n}D}\geq \frac{\left(1-\rho \right)}{\eta \rho },\frac{\gamma_{th}\gamma_{R_{n}P}}{\Psi }<\frac{\left(1-\rho \right)}{\eta \rho }\right\}\\ = & \mathrm{Pr}\left\{X\geq \frac{\gamma_{th}}{(1-\rho )\Psi }\right\}\times \mathrm{Pr}\left\{\gamma_{R_{n}D}\geq \frac{\left(1-\rho \right)}{\eta \rho }\right\}\times \mathrm{Pr}\left\{\gamma_{R_{n}P}<\frac{\Psi (1-\rho )}{\eta \rho \gamma_{th}}\right\}\\ = & \left\{1-F_{X}\left(\frac{\gamma_{th}}{(1-\rho )\Psi }\right)\right\}\times \left\{1-F_{\gamma_{R_{n}D}}\left(\frac{\left(1-\rho \right)}{\eta \rho }\right)\right\}\times F_{\gamma_{R_{n}P}}\left(\frac{\Psi (1-\rho )}{\eta \rho \gamma_{th}}\right),\\ \overset{\left(a\right)}{=} & \sum_{k=1}^{M}\frac{{\left(-1\right)}^{k+1}C_{M}^{k}\lambda_{\mathrm{SP}}\left(1-\rho \right)\Psi }{k\lambda_{\mathrm{SR}}\gamma_{th}+\lambda_{\mathrm{SP}}\left(1-\rho \right)\Psi }\exp\left(-\lambda_{\mathrm{RD}}\zeta \right)\left\{1-\exp\left(-\frac{\lambda_{\mathrm{RP}}\Psi \zeta }{\gamma_{th}}\right)\right\}. \end{array}$$

Here, $\left(a\right)$ is held by employing the results of (22). Having $\Xi_{11}$ and $\Xi_{12}$ in hand, $\Xi_{1}$ in (20) is then computed as

(26) $$\begin{array}{cc} \Xi_{1}= & \sum_{k=1}^{M}\frac{{\left(-1\right)}^{k+1}C_{M}^{k}\lambda_{\mathrm{SP}}\left(1-\rho \right)\Psi }{k\lambda_{\mathrm{SR}}\gamma_{th}+\lambda_{\mathrm{SP}}\left(1-\rho \right)\Psi }\left[\exp\left(-\frac{\zeta \Psi \left[\lambda_{\mathrm{RP}}+\lambda_{\mathrm{RD}}\right]}{\gamma_{th}}\right)\right.\\ \; & \left.-\frac{\exp\left(-\frac{\zeta \Psi }{\gamma_{th}}\left[\lambda_{\mathrm{RD}}+\frac{\Psi \lambda_{\mathrm{RP}}}{\gamma_{th}}\right]\right)}{\left(1+\frac{\Psi \lambda_{\mathrm{RP}}}{\gamma_{th}\lambda_{\mathrm{RD}}}\right)}+\exp\left(-\lambda_{\mathrm{RD}}\zeta \right)-\exp\left(-\zeta \left[\lambda_{\mathrm{RD}}+\frac{\lambda_{\mathrm{RP}}\Psi }{\gamma_{th}}\right]\right)\right]. \end{array}$$

After obtaining $\Xi_{1}$, we now compute $\Xi_{2}$ in (19) as follows:

(27) $$\begin{array}{cc} \Xi_{2}= & \mathrm{Pr}\left\{X\geq \frac{\gamma_{th}}{\Psi \eta \rho \gamma_{R_{n}D}},\gamma_{R_{n}D}\geq \frac{\gamma_{th}\gamma_{R_{n}P}}{\Psi },\frac{\gamma_{th}}{(1-\rho )\Psi }<\frac{\gamma_{th}}{\Psi \eta \rho \gamma_{R_{n}D}}\right\}\\ = & \mathrm{Pr}\left\{X\geq \frac{\gamma_{th}}{\Psi \eta \rho \gamma_{R_{n}D}},\frac{\gamma_{th}\gamma_{R_{n}P}}{\Psi }\leq \gamma_{R_{n}D}<\zeta \right\}\\ = & \int_{0}^{\frac{\zeta \Psi }{\gamma_{th}}}f_{\gamma_{R_{n}P}}\left(x\right)dx\int_{\frac{\gamma_{th}x}{\Psi }}^{\zeta }f_{\gamma_{R_{n}D}}\left(y\right)dy\int_{\frac{\gamma_{th}}{\Psi \eta \rho y}}^{\infty }f_{X}\left(z\right)dz,\\ \overset{\left(a\right)}{=} & \sum_{k=1}^{M}{\left(-1\right)}^{k+1}C_{M}^{k}\int_{0}^{\frac{\zeta \Psi }{\gamma_{th}}}f_{\gamma_{R_{n}P}}\left(x\right)dx\underset{\Xi_{21}}{\underbrace{\int_{\frac{\gamma_{th}x}{\Psi }}^{\zeta }\frac{\lambda_{\mathrm{SP}}\lambda_{\mathrm{RD}}\Psi \eta \rho y}{k\lambda_{\mathrm{SR}}\gamma_{th}+\lambda_{\mathrm{SP}}\Psi \eta \rho y}\exp\left(-\lambda_{\mathrm{RD}}y\right)dy}} \end{array}$$

where $\left(a\right)$ is held with the help of Lemma 1. By employing integration by parts, $\Xi_{21}$ is evaluated as

(28) $$\begin{array}{cc} \Xi_{21}= & \left(\frac{\frac{\gamma_{th}\lambda_{\mathrm{RD}}x}{\Psi }\exp\left(-\frac{\gamma_{th}\lambda_{\mathrm{RD}}x}{\Psi }\right)}{\frac{\gamma_{th}\lambda_{\mathrm{RD}}x}{\Psi }+\frac{k\lambda_{\mathrm{SR}}\lambda_{\mathrm{RD}}\gamma_{th}}{\lambda_{\mathrm{SP}}\Psi \eta \rho }}\right)-\left(\frac{\lambda_{\mathrm{RD}}\zeta \exp\left(-\lambda_{\mathrm{RD}}\zeta \right)}{\lambda_{\mathrm{RD}}\zeta +\frac{k\lambda_{\mathrm{SR}}\lambda_{\mathrm{RD}}\gamma_{th}}{\lambda_{\mathrm{SP}}\Psi \eta \rho }}\right)\\ \; & +\underset{\Xi_{22}}{\underbrace{\frac{k\lambda_{\mathrm{SR}}\gamma_{th}}{\lambda_{\mathrm{SP}}\Psi \eta \rho }\int_{\frac{\gamma_{th}x}{\Psi }}^{\zeta }\frac{\exp\left(-\lambda_{\mathrm{RD}}y\right)}{{\left(y+\frac{k\lambda_{\mathrm{SR}}\gamma_{th}}{\lambda_{\mathrm{SP}}\Psi \eta \rho }\right)}^{2}}dy}}\\ \overset{\left(a\right)}{=} & \frac{\frac{\gamma_{th}\lambda_{\mathrm{RD}}x}{\Psi }\exp\left(-\frac{\gamma_{th}\lambda_{\mathrm{RD}}x}{\Psi }\right)}{\frac{\gamma_{th}\lambda_{\mathrm{RD}}x}{\Psi }+\frac{k\lambda_{\mathrm{SR}}\lambda_{\mathrm{RD}}\gamma_{th}}{\lambda_{\mathrm{SP}}\Psi \eta \rho }}-\frac{\lambda_{\mathrm{RD}}\zeta \exp\left(-\lambda_{\mathrm{RD}}\zeta \right)}{\lambda_{\mathrm{RD}}\zeta +\frac{k\lambda_{\mathrm{SR}}\lambda_{\mathrm{RD}}\gamma_{th}}{\lambda_{\mathrm{SP}}\Psi \eta \rho }}\\ \; & +\frac{k\lambda_{\mathrm{SR}}\gamma_{th}}{\lambda_{\mathrm{SP}}\Psi \eta \rho }\exp\left(-\frac{\gamma_{th}\lambda_{\mathrm{RD}}x}{\Psi }\right)\int_{0}^{\zeta -\frac{\gamma_{th}x}{\Psi }}\frac{\exp\left(-\lambda_{\mathrm{RD}}t\right)}{{\left(t+\frac{\gamma_{th}x}{\Psi }+\frac{k\lambda_{\mathrm{SR}}\gamma_{th}}{\lambda_{\mathrm{SP}}\Psi \eta \rho }\right)}^{2}}dt\\ \overset{\left(b\right)}{=} & \frac{\frac{\gamma_{th}\lambda_{\mathrm{RD}}x}{\Psi }\exp\left(-\frac{\gamma_{th}\lambda_{\mathrm{RD}}x}{\Psi }\right)}{\frac{\gamma_{th}\lambda_{\mathrm{RD}}x}{\Psi }+\frac{k\lambda_{\mathrm{SR}}\lambda_{\mathrm{RD}}\gamma_{th}}{\lambda_{\mathrm{SP}}\Psi \eta \rho }}-\frac{\lambda_{\mathrm{RD}}\zeta \exp\left(-\lambda_{\mathrm{RD}}\zeta \right)}{\lambda_{\mathrm{RD}}\zeta +\frac{k\lambda_{\mathrm{SR}}\lambda_{\mathrm{RD}}\gamma_{th}}{\lambda_{\mathrm{SP}}\Psi \eta \rho }}\\ \; & +\mu \exp\left(\mu \right)\left[\Gamma \left(-1,\frac{\gamma_{th}\lambda_{\mathrm{RD}}x}{\Psi }+\mu \right)-\Gamma \left(-1,\lambda_{\mathrm{RD}}\zeta +\mu \right)\right], \end{array}$$

By changing variable $t=y-\frac{\gamma_{th}x}{\Psi }$, we obtain $\left(a\right)$; $\left(b\right)$ is the outcome of ([41], 3.462.17), $\mu =\frac{k\lambda_{\mathrm{SR}}\lambda_{\mathrm{RD}}\gamma_{th}}{\lambda_{\mathrm{SP}}\Psi \eta \rho }$, and $\Gamma \left(\alpha ,x\right)=\int_{x}^{\infty }e^{-t}t^{\alpha -1}dt$ is the incomplete gamma function.

Next, substituting (28) into (27), $\Xi_{2}$ can be recomputed as

(29) $$\begin{array}{cc} \Xi_{2}= & \sum_{k=1}^{M}{\left(-1\right)}^{k+1}C_{M}^{k}\lambda_{\mathrm{RP}}\int_{0}^{\frac{\zeta \Psi }{\gamma_{th}}}\exp\left(-\lambda_{\mathrm{RP}}x\right)dx\\ \; & \times \left[\frac{\frac{\gamma_{th}\lambda_{\mathrm{RD}}x}{\Psi }\exp\left(-\frac{\gamma_{th}\lambda_{\mathrm{RD}}x}{\Psi }\right)}{\frac{\gamma_{th}\lambda_{\mathrm{RD}}x}{\Psi }+\frac{k\lambda_{\mathrm{SR}}\lambda_{\mathrm{RD}}\gamma_{th}}{\lambda_{\mathrm{SP}}\Psi \eta \rho }}-\frac{\lambda_{\mathrm{RD}}\zeta \exp\left(-\lambda_{\mathrm{RD}}\zeta \right)}{\lambda_{\mathrm{RD}}\zeta +\frac{k\lambda_{\mathrm{SR}}\lambda_{\mathrm{RD}}\gamma_{th}}{\lambda_{\mathrm{SP}}\Psi \eta \rho }}\right.\\ \; & \left.+\mu \exp\left(\mu \right)\left[\Gamma \left(-1,\frac{\gamma_{th}\lambda_{\mathrm{RD}}x}{\Psi }+\mu \right)-\Gamma \left(-1,\lambda_{\mathrm{RD}}\zeta +\mu \right)\right]\right]\\ = & \underset{\Xi_{23}}{\underbrace{\sum_{k=1}^{M}{\left(-1\right)}^{k+1}C_{M}^{k}\lambda_{\mathrm{RP}}\int_{0}^{\frac{\zeta \Psi }{\gamma_{th}}}\frac{x\exp\left(-x\left[\lambda_{\mathrm{RP}}+\frac{\gamma_{th}\lambda_{\mathrm{RD}}}{\Psi }\right]\right)}{x+\frac{\mu \Psi }{\gamma_{th}\lambda_{\mathrm{RD}}}}dx}}\\ \; & -\sum_{k=1}^{M}\frac{{\left(-1\right)}^{k+1}C_{M}^{k}\lambda_{\mathrm{RP}}\lambda_{\mathrm{RD}}\zeta \exp\left(-\lambda_{\mathrm{RD}}\zeta \right)}{\lambda_{\mathrm{RD}}\zeta +\mu }\times \left(1-\exp\left(-\frac{\lambda_{\mathrm{RP}}\zeta \Psi }{\gamma_{th}}\right)\right)\\ \; & +\underset{\Xi_{24}}{\underbrace{\sum_{k=1}^{M}{\left(-1\right)}^{k+1}C_{M}^{k}\lambda_{\mathrm{RP}}\mu \exp\left(\mu \right)\int_{0}^{\frac{\zeta \Psi }{\gamma_{th}}}\exp\left(-\lambda_{\mathrm{RP}}x\right)\times \Gamma \left(-1,\frac{\gamma_{th}\lambda_{\mathrm{RD}}x}{\Psi }+\mu \right)dx}}\\ \; & -\sum_{k=1}^{M}{\left(-1\right)}^{k+1}C_{M}^{k}\lambda_{\mathrm{RP}}\mu \exp\left(\mu \right)\Gamma \left(-1,\lambda_{\mathrm{RD}}\zeta +\mu \right)\times \left(1-\exp\left(-\frac{\lambda_{\mathrm{RP}}\zeta \Psi }{\gamma_{th}}\right)\right). \end{array}$$

Investigating (29), we observe that $\Xi_{24}$, unfortunately, is not able to be computed in closed-form expression due to the fact that the upper limit of the integration does not approach infinity as well as the generality of the incomplete Gamma function. It, however, is effortlessly computed by employing a numerical method with the help of several commercial software such as Matlab, Python, and Mathematica. Moreover, contrary to $\Xi_{24}$, $\Xi_{23}$ can be evaluated in the closed-form expression by deploying the integration by parts and the assistance of ([41], 3.462.17) as follows:

(30) $$\begin{array}{cc} \Xi_{23}= & \sum_{k=1}^{M}{\left(-1\right)}^{k+1}C_{M}^{k}\lambda_{\mathrm{RP}}\left[\exp\left(\mu +\frac{\lambda_{\mathrm{RP}}\mu \Psi }{\gamma_{th}\lambda_{\mathrm{RD}}}\right)\left[\Gamma \left(-1,\mu +\frac{\lambda_{\mathrm{RP}}\mu \Psi }{\gamma_{th}\lambda_{\mathrm{RD}}}\right)\right.\right.\\ \; & \left.\left.-\Gamma \left(-1,\mu +\lambda_{\mathrm{RD}}\zeta +\frac{\lambda_{\mathrm{RP}}\Psi }{\gamma_{th}}\left(\zeta +\frac{\mu }{\lambda_{\mathrm{RD}}}\right)\right)\right]-\frac{\exp\left(-\frac{\lambda_{\mathrm{RP}}\zeta \Psi }{\gamma_{th}}-\lambda_{\mathrm{RD}}\zeta \right)}{\left(1+\frac{\mu }{\lambda_{\mathrm{RD}}\zeta }\right)\left(\lambda_{\mathrm{RP}}+\frac{\gamma_{th}\lambda_{\mathrm{RD}}}{\Psi }\right)}\right]. \end{array}$$

Finally, substituting (26) and (29) into (19), OP defined in (14) is computed as

(31) $$\begin{array}{cc} \mathrm{OP}= & 1-\Xi_{1}-\Xi_{2},\\ \Xi_{1}= & \sum_{k=1}^{M}\frac{{\left(-1\right)}^{k+1}C_{M}^{k}\lambda_{\mathrm{SP}}\left(1-\rho \right)\Psi }{k\lambda_{\mathrm{SR}}\gamma_{th}+\lambda_{\mathrm{SP}}\left(1-\rho \right)\Psi }\left[\exp\left(-\frac{\zeta \Psi \left[\lambda_{\mathrm{RP}}+\lambda_{\mathrm{RD}}\right]}{\gamma_{th}}\right)\right.\\ \; & \left.-\frac{\exp\left(-\frac{\zeta \Psi }{\gamma_{th}}\left[\lambda_{\mathrm{RD}}+\frac{\Psi \lambda_{\mathrm{RP}}}{\gamma_{th}}\right]\right)}{\left(1+\frac{\Psi \lambda_{\mathrm{RP}}}{\gamma_{th}\lambda_{\mathrm{RD}}}\right)}+\exp\left(-\lambda_{\mathrm{RD}}\zeta \right)-\exp\left(-\zeta \left[\lambda_{\mathrm{RD}}+\frac{\lambda_{\mathrm{RP}}\Psi }{\gamma_{th}}\right]\right)\right]\\ \Xi_{2}= & \sum_{k=1}^{M}{\left(-1\right)}^{k+1}C_{M}^{k}\lambda_{\mathrm{RP}}\left[\exp\left(\mu +\frac{\lambda_{\mathrm{RP}}\mu \Psi }{\gamma_{th}\lambda_{\mathrm{RD}}}\right)\left[\Gamma \left(-1,\mu +\frac{\lambda_{\mathrm{RP}}\mu \Psi }{\gamma_{th}\lambda_{\mathrm{RD}}}\right)\right.\right.\\ \; & \left.\left.-\Gamma \left(-1,\mu +\lambda_{\mathrm{RD}}\zeta +\frac{\lambda_{\mathrm{RP}}\Psi }{\gamma_{th}}\left(\zeta +\frac{\mu }{\lambda_{\mathrm{RD}}}\right)\right)\right]-\frac{\exp\left(-\frac{\lambda_{\mathrm{RP}}\zeta \Psi }{\gamma_{th}}-\lambda_{\mathrm{RD}}\zeta \right)}{\left(1+\frac{\mu }{\lambda_{\mathrm{RD}}\zeta }\right)\left(\lambda_{\mathrm{RP}}+\frac{\gamma_{th}\lambda_{\mathrm{RD}}}{\Psi }\right)}\right]\\ \; & -\sum_{k=1}^{M}\frac{{\left(-1\right)}^{k+1}C_{M}^{k}\lambda_{\mathrm{RP}}\lambda_{\mathrm{RD}}\zeta \exp\left(-\lambda_{\mathrm{RD}}\zeta \right)}{\lambda_{\mathrm{RD}}\zeta +\mu }\times \left(1-\exp\left(-\frac{\lambda_{\mathrm{RP}}\zeta \Psi }{\gamma_{th}}\right)\right)\\ \; & +\sum_{k=1}^{M}{\left(-1\right)}^{k+1}C_{M}^{k}\lambda_{\mathrm{RP}}\mu \exp\left(\mu \right)\int_{0}^{\frac{\zeta \Psi }{\gamma_{th}}}\exp\left(-\lambda_{\mathrm{RP}}x\right)\times \Gamma \left(-1,\frac{\gamma_{th}\lambda_{\mathrm{RD}}x}{\Psi }+\mu \right)dx\\ \; & -\sum_{k=1}^{M}{\left(-1\right)}^{k+1}C_{M}^{k}\lambda_{\mathrm{RP}}\mu \exp\left(\mu \right)\Gamma \left(-1,\lambda_{\mathrm{RD}}\zeta +\mu \right)\times \left(1-\exp\left(-\frac{\lambda_{\mathrm{RP}}\zeta \Psi }{\gamma_{th}}\right)\right). \end{array}$$

## 4. Simulation Results

In this section, computer-based simulation results are given to verify the correctness of the derived mathematical framework and to discover the behaviors of OP with respect to several essential system parameters, namely, the transmit power, the number of relays, the power splitting ratio, and so forth. In particular, the Monte Carlo method is utilized, and the number of realizations is $10^{6}$ to avoid fluctuation due to insufficient samples. Unless otherwise stated, the following parameters are applied: β=2, η=0.8, ρ = 0.5, Ψ = 5 dB, and R = 0.5 bps/Hz. All nodes are located on the two-dimensional plane where the source is fixed at the origin, i.e., (0, 0), the positions of other nodes, i.e., relay $R_{n}$, destination D, and primary P, are (1, 0), (1.5, 0), and (1, 1), respectively.

**Figure 3:**
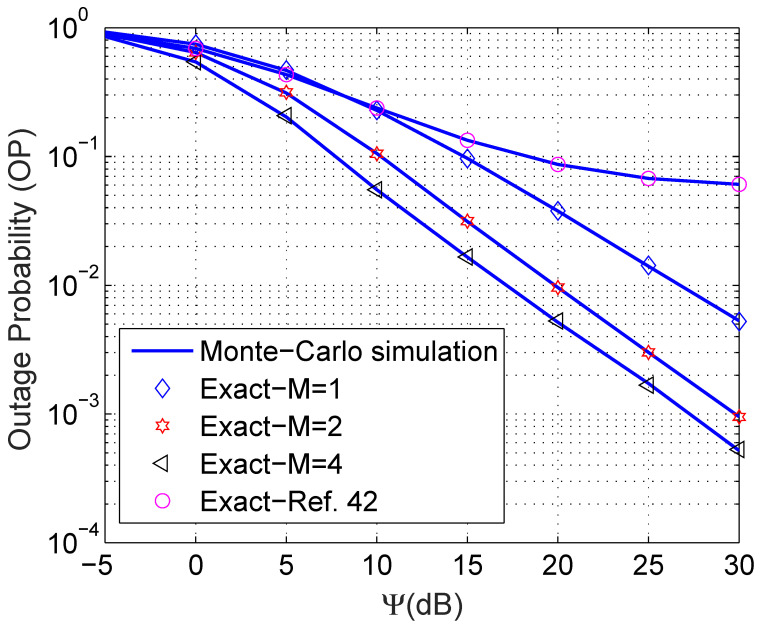
Outage probability versus Ψ (dB) with η=0.8, ρ = 0.5, and R = 0.5 bps/Hz.

Figure 3 shows the impact of the interference power over noise variance $\Psi =\frac{I_{p}}{N_{0}}$ on the performance of the OP. The proposed mathematical framework absolutely matches the Monte Carlo simulations. Moreover, we observe that increasing the number of relays *M* obviously improves the OP. To be more precise, with Ψ=30 dB, the OP of M=1 is approximately $5\times 10^{-3}$, while the OP of M=4 is 10× better and is around $5\times 10^{-4}$. Additionally, the proposed framework outperforms work presented in the literature [42]. In particular, the OP of Ref. [42] only achieves around 0.06 when Ψ=30, while the OP of the proposed scheme with M=1 is already approximately 0.005. In addition, the pace of improvement when Ψ is small and large is different. In particular, the OP dramatically improves when Ψ is small and a fair improvement is experienced when Ψ is large. The main reason behind this phenomenon is that when Ψ is sufficiently large, the outage event almost disappears, as a consequence, a slight enhancement is observed.

**Figure 4:**
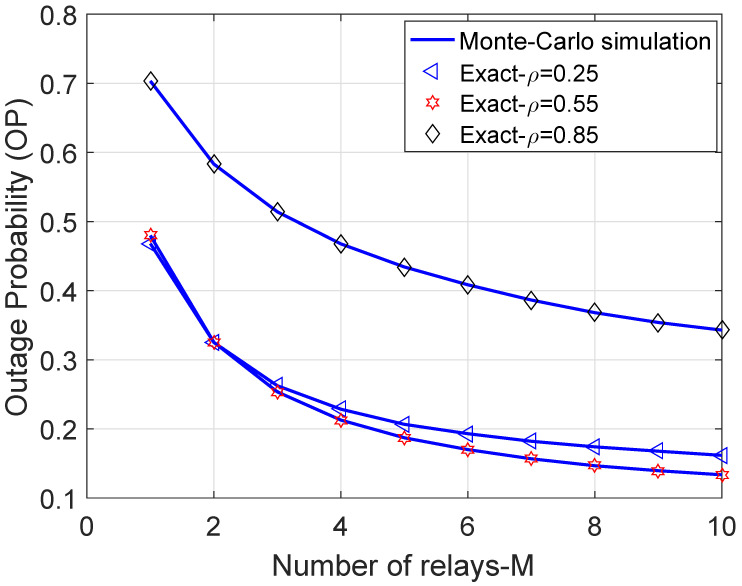
Outage probability versus number of relays-M with η = 0.8, R = 0.5 bps/Hz, and Ψ = 5 dB.

Figure 4 shows the outage probability versus the number of relays *M*. As mentioned in Figure 3, scaling up *M* certainly ameliorates the OP. Nevertheless, the benefits of increasing *M* are not the same when *M* is limited versus *M* as large. More precisely, when M<4, OP declines exponentially, while when M≥4, OP fairly decreases. Figure 4 confirms again the accuracy of the derived mathematical framework.

**Figure 5:**
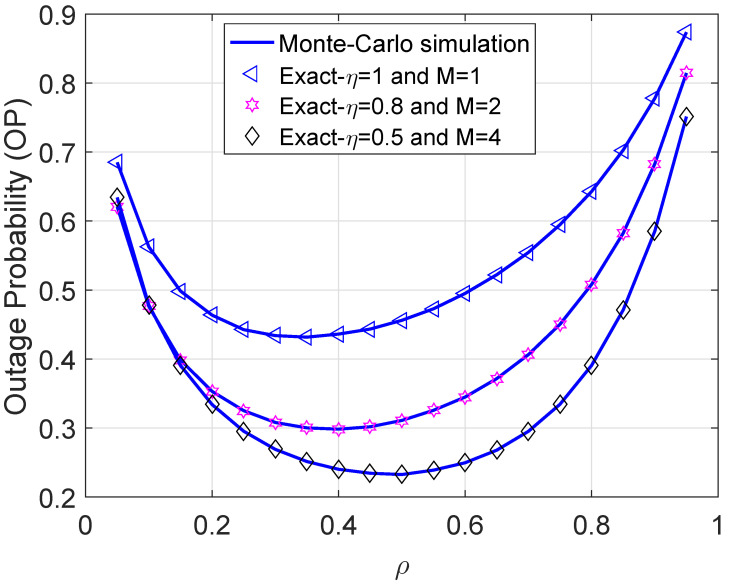
Outage probability versus ρ with R = 0.5 bps/Hz and Ψ = 5 dB.

We investigate the behavior of OP regarding the power splitting ratio ρ in Figure 5. We see that OP first plunges with ρ after reaching its minimum, OP then turns and constantly rises when ρ moves from 0 to 1. When ρ is small, the amount of harvested energy at R is limited, which leads to lower SNR thereby degrading system performance. When ρ is close to 1, on the other hand, the received signals being added into the information decoder of R are limited too, hence, scaling up the OP. In addition, the curve with M=4 is not always superior to the others for all values of ρ. In particular, when ρ is small, the curve with M=2 is better than the case of M=4. However, in general, increasing the number of relays can overcome the constraint of the hardware limitation. Furthermore, the optimal value of ρ denoted by $\rho^{*}$ where OP achieves its minimum can be straightforwardly derived from this figure. For example, the $\rho^{*}$ of curve with η=0.5 and M=4 is approximately 0.5, while the $\rho^{*}$ of the curve with η=0.8 and M=2 is just below 0.4.

**Figure 6:**
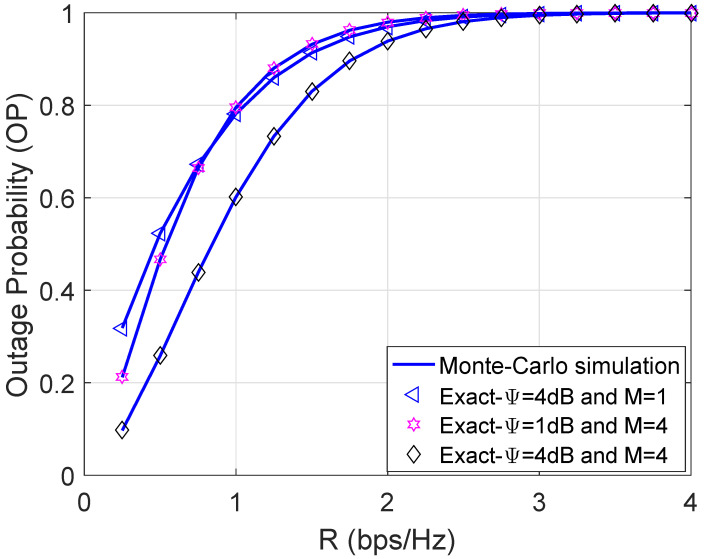
Outage probability versus R with η=0.8 and ρ = 0.5.

Figure 6 stretches the OP versus the targeted rate R. There is no doubt that the larger the R the higher the OP. We can directly explain this trend by yielding the definition of the OP. Interestingly, the curve with M=4 and Ψ=1 dB does not consistently outperform case M=1 and Ψ=4 dB. This means that we can ameliorate the system performance either by increasing the number of relay nodes or scaling up the transmit power.

**Figure 7:**
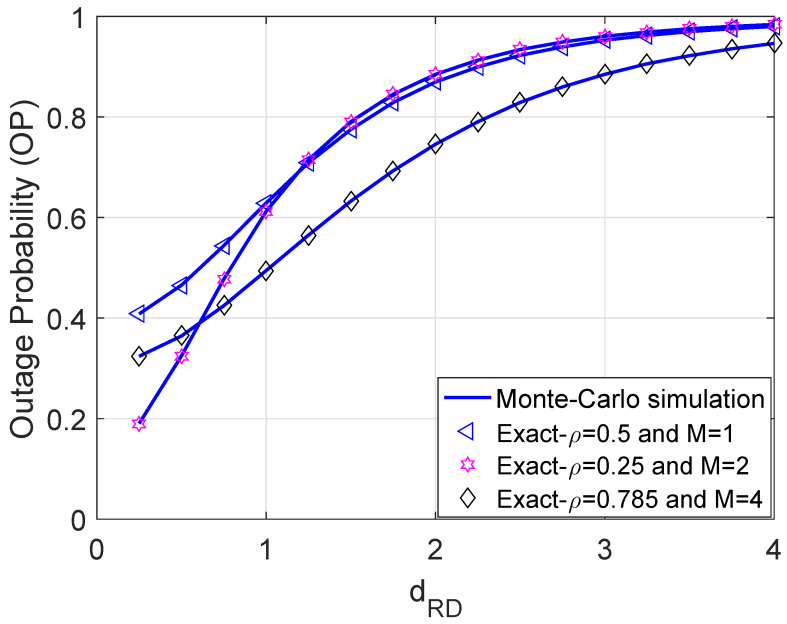
Outage probability versus $d_{\mathrm{RD}}$ with η = 0.8, R = 0.5 bps/Hz, and Ψ = 5 dB.

Figure 7 addresses the impact of the mobility of the destination on the performance of the considered networks. To be more precise, destination D is moving from R to far away. The further the $d_{\mathrm{RD}}$ is, the worse the OP. The rationale behind this trend is that increasing $d_{\mathrm{RD}}$ is directly proportional to the large-scale path loss; thus, this deteriorates the channel gain and increases the OP. Moreover, the OP performance can be improved significantly by properly optimizing the power splitting ratio. In particular, we observe that the performance of curves with M=1 and M=2 is more or less equivalent unless the $d_{\mathrm{RD}}$ approaches zero.

## 5. Conclusions

The outage performance of the SWIPT-based cognitive radio networks was addressed in this paper. In particular, all relay nodes were equipped not only with a conventional information decoder but also an energy harvester, so that they were able to concurrently receive information and harvest the energy from the incoming signals. Additionally, the opportunistic relaying was taken into account to both ameliorate the outage probability and save the consumed resources. The numerical results illustrated that either increasing the number of relay nodes or raising the transmit power was beneficial for the considered networks. The paper can be extended in several directions: (i) We can replace the fixed relay by high mobility unmanned aerial vehicles (UAVs) devices [43,44] or employ advanced meta material technology to enhance energy efficiency, i.e., the reconfigurable intelligent surfaces (RIS) [45,46]; (ii) Diversity combining at the destination, i.e., maximal ratio combining or selection combining, would also be considered a simple solution to raise the system’s diversity gain [47,48]; (iii) Consideration of the networks could extend to investigating the security aspect as well [49,50]; and (iv) Two-way relaying or full-duplex relaying would be a feasible and interesting extension as well [51,52].
